# Early Affective Processing in Patients with Acute Posttraumatic Stress Disorder: Magnetoencephalographic Correlates

**DOI:** 10.1371/journal.pone.0071289

**Published:** 2013-08-19

**Authors:** Markus Burgmer, Maimu Alissa Rehbein, Marco Wrenger, Judith Kandil, Gereon Heuft, Christian Steinberg, Bettina Pfleiderer, Markus Junghöfer

**Affiliations:** 1 Institute for Biomagnetism and Biosignalanalysis, University Hospital of Münster, Münster, Germany; 2 Department of Psychosomatics and Psychotherapy, University Hospital of Münster, Münster, Germany; 3 Asklepios Hospital Teupitz, Psychiatry and Psychotherapy, Teupitz, Germany; 4 Department of Clinical Radiology, University Hospital of Münster, Münster, Germany; University of Wuerzburg, Germany

## Abstract

**Background:**

In chronic PTSD, a preattentive neural alarm system responds rapidly to emotional information, leading to increased prefrontal cortex (PFC) activation at early processing stages (<100 ms). Enhanced PFC responses are followed by a reduction in occipito-temporal activity during later processing stages. However, it remains unknown if this neuronal pattern is a result of a long lasting mental disorder or if it represents changes in brain function as direct consequences of severe trauma.

**Methodology:**

The present study investigates early fear network activity in acutely traumatized patients with PTSD. It focuses on the question whether dysfunctions previously observed in chronic PTSD patients are already present shortly after trauma exposure. We recorded neuromagnetic activity towards emotional pictures in seven acutely traumatized PTSD patients between one and seven weeks after trauma exposure and compared brain responses to a balanced healthy control sample. Inverse modelling served for mapping sources of differential activation in the brain.

**Principal Findings:**

Compared to the control group, acutely traumatized PTSD patients showed an enhanced PFC response to high-arousing pictures between 60 to 80 ms. This rapid prefrontal hypervigilance towards arousing pictorial stimuli was sustained during 120–300 ms, where it was accompanied by a reduced affective modulation of occipito-temporal neural processing.

**Conclusions:**

Our findings indicate that the hypervigilance-avoidance pattern seen in chronic PTSD is not necessarily a product of an endured mental disorder, but arises as an almost immediate result of severe traumatisation. Thus, traumatic experiences can influence emotion processing strongly, leading to long-lasting changes in trauma network activation and expediting a chronic manifestation of maladaptive cognitive and behavioral symptoms.

## Introduction

Post-traumatic stress disorder (PTSD) is a severe mental disorder that can occur after the experience of a highly stressful or traumatic event. Its core characteristics are hyperarousal, behavioral avoidance and/or emotional numbing, and frequent intrusions of traumatic material into consciousness [1], [2]. Among others [3], one theoretical approach explains the pathology of PTSD by drawing on the nature of the traumatic event itself and its storage in a fear structure [4], [5]. All aspects of the trauma, including cognitions, emotions, sensory, and physiological responses, are stored as ‘nodes’ in a fear network, which provides information templates for quickly activating a fear response. Due to spreading activation between nodes, excitement of a single element is sufficient to activate the whole network and to initiate a fear reaction. With repeated exposure to stressful experiences, elements are added and connections between different nodes are established or strengthened by ways of Hebbian learning, so that the activation threshold of the fear structure is lowered [6], [7]. As a result, fear-related stimuli may readily excite the fear network, which, in turn, can trigger a fear response and interrupt on-going cognitive mechanisms [8].

Several functional brain imaging studies revealed relevant brain areas involved in the trauma network by investigating brain responses to emotional stimuli in PTSD patients. For example, amygdala activation was enhanced towards trauma-related [9], [10] and not trauma-related, emotional information [11], [12]. Furthermore, disturbed prefrontal cortex (PFC) function was recorded during the processing of emotional information. More specifically, both hypoactivation [12],[13] and hyperactivation [14] within the medial prefrontal cortex, including orbitofrontal areas and the anterior cingulate cortex, were reported [15].

Due to their excellent temporal resolution, event-related potential or magnetic field (ERP, ERF) studies can reveal the temporal dynamics of disturbed information processing in PTSD and, thus, complement the view of hemodynamic functional imaging studies. Indeed, ERP studies using auditory stimuli found alterations in stimulus processing already at 50 ms after stimulus onset pointing towards impaired sensory gating processes in PTSD [16]–[18]. In vision, altered processing of trauma-related and not trauma-related emotional material was reported as early as 100 ms post-stimulus [19]–[21] and also later processing stages, such as the P200 [22], [23] and P300 [19], [24], seem to be affected. However, results about altered ERP-amplitudes in vision appear rather inconsistent, since both augmented and attenuated amplitudes have been reported [25], [26].

Recent whole-head ERF-studies using distributed source models may provide a solid explanation to these mixed results. Indeed, both decreased and increased steady-state responses towards negative pictures were recorded in PTSD, but the first stemmed from occipital areas and the latter from superior parietal areas [27]. This biphasic pattern was confirmed by findings showing a decrease in affective modulation of parieto-occipital areas during 206–256 ms, preceded by enhanced PFC activation towards negative pictures [28]. It was concluded that such heightened PFC responsiveness displays a rapid threat detection mechanism activating a primary orienting response towards threatening stimuli, while the reduced responses in sensory areas reflect a subsequent avoidance reaction. Further support for such a hypervigilance-avoidance hypothesis (cf. 28) in PTSD is provided by another ERF-study [6] showing enhanced PFC activation during 60 to 80 ms after stimulus onset and suppressed responses in occipital areas during 100 to 300 ms, consistent with the interval of the early posterior negativity (EPN) [29] or the EPN-m as magnetic counterpart respectively [30].

However, it remains unclear if this hypervigilance-avoidance pattern in PTSD arises as a consequence of long-term chronic stress and a frequently developed comorbid depression or if it is verifiable already in the acute phase of PTSD. So far, the number of both hemodynamic functional imaging [31]–[33] and electrophysiological [34] studies examining acutely traumatized patients is still quite low. Thus, even though the first [31]–[33] indicate alterations of amygdala and PFC activation immediately after trauma and the second [34] suggest changes in information processing at later processing stages shortly after trauma exposure, acute effects of trauma on brain and especially rapid brain functions remain unclear.

Here, we aim at clarifying the question whether the hypervigilance-avoidance pattern seen in patients with chronic PTSD would already be present in acutely traumatized patients with PTSD. We hypothesize that patients as compared to controls would exhibit decreased affective modulation in the occipital cortex during the EPN-m, while showing very early (<100 ms) exaggerated PFC responsiveness towards emotional stimuli.

## Methods and Materials

### Participants

Seven (5 female, all Caucasian) victims of physical assault or violence (median age: 42 years, range: 18–63) were recruited consecutively from the trauma ambulance of the Department of Psychosomatics and Psychotherapy, University Hospital Muenster, Germany. Following prevention requirements, police officers had suggested them to visit the trauma ambulance after they had suffered man-made physical assault or violence. Thus, patients were all walk-ins and were not recruited especially for the study. In accordance with the DSM-IV all patients were considered to suffer from acute PTSD if the symptoms had been present for less than three months. Thus, patients were only included in the study if the traumatic event had occurred not longer than 90 days before the visit. All walk-in patients indicated that the traumatic event had taken place between 13 and 52 days (mean: 25 days) before seeking treatment. They had never before encountered any traumatic events and a psychiatric examination with a standardized interview (SCID I, SCID II) did not show a history of mental or personality disorders.

Seven healthy controls were matched in sex, age, and schooling. Controls did not show symptoms of mental disorder (SCID I screening tool) and did not report any traumatic experiences. All participants were right handed (Edingburgh Handedness Inventory) [35], had normal or corrected-to-normal vision, and were not taking psychopharmacological medication during the time of the study. All participants provided written informed consent and the local ethics committee (University of Muenster) approved the protocol (2006-011-f-S). Potential participants who declined to participate or otherwise did not participate were eligible for treatment and were not disadvantaged in any other way by not participating in the study.

### Psychological assessment

In addition to the structured clinical interviews (SCID I, SCID II), the Clinical Administered PTSD Scale (CAPS) was performed with all patients to guarantee valid PTSD diagnosis. The CAPS is a reliable and valid tool for assessing core and associated symptoms of current and lifetime PTSD [36]. It quantifies symptom frequency and severity for each PTSD diagnostic criterion of DSM-IV (Criteria A-D) and a diagnosis is made if all 4 criteria are fulfilled. Because we wanted to include patients as early as possible after the trauma exposure patients were diagnosed as PTSD even they did not fulfil the time criterion of 4 weeks symptom duration (Criterion E). In addition, patients completed German versions of the Impact of Event Scale revised edition (IES-R) [37] and the Hamilton Anxiety and Depression Scale (HADS) [38].

### Stimulus material

Based on normative ratings of emotional valence and arousal, 500 pictures from the International Affective Picture System (IAPS) [39] were divided into five picture categories – high arousal positive (HiPos erotica, sports, etc.), low arousal positive (LoPos: family, landscapes, etc.), neutral (Neut: people, road traffic, etc.), low arousal negative (LoNeg: illness, pollution, etc.), and high arousal negative (HiNeg: attack, mutilation, etc.) – with 100 pictures per category. Statistical tests revealed that critical picture parameters such as brightness, contrast, color distribution, and complexity did not differ across picture categories.

### MEG data-acquisition and data-analysis

All pictures were presented once in a perceptual random order as a continuous stream for 660 ms each [40]. Participants were asked to passively view the stimuli while keeping their eyes focused on an overlaid fixation cross. Visual evoked magnetic fields were acquired using a 275 MEG whole-head sensor system (VSM Medtech Ltd.). Landmark coils attached to the auditory canals and the nasion monitored the participant's position in the MEG scanner. The averaged movements of participants during the MEG session (Euclidian distances to the start position averaged across all time points and across the three landmark coils) laid between 0.7 and 2.2 mm in the patients and between 0.3 and 2.7 mm in the control group and did not differ across groups. Individual head shape information was acquired by structural MRI. Continuous MEG data in the frequency band between 0 and 150 Hz were recorded with a sampling rate of 600 Hz.

MEG data analysis was conducted with the EMEGS software (www.emegs.org) [41]. Responses were filtered offline with a zero-phase (forward/backward) Butterworth second order high-pass filter with 0.1 Hz cut-off value and a zero-phase Butterworth fourth order low-pass filter with 48 Hz cut-off value. The filtered responses were aligned to stimulus onset with an averaging epoch ranging from 200 ms before to 600 ms after stimulus presentation. Epochs were baseline-adjusted using a 150 ms pre-stimulus interval, which lasted from 150 ms before stimulus presentation until stimulus onset. A method for statistical control of artifacts in high density EEG/MEG data was used for single trial data editing and artifact rejection [cf. 42]. Trials showing artifacts at too many sensors were removed, resulting in a mean number of trials of 450.3 (SD  = 26.5) for patients and 459.4 (SD  = 21.1) for controls (out of originally 500 trials). Channels contaminated by artifacts were interpolated using weighted spherical splines fit to all remaining sensors. The mean number of interpolated channels was 2.31 (SD  = 1.36) for patients and 2.73 (SD  = 0.85) for controls. Univariate Analysis of Variance (ANOVA) testing the effect of GROUP (patients, controls) on rejected trials and interpolated sensors indicated that patients and controls differed neither in the final number of trials (F(1,12)  = 0.51, n.s.) nor in the mean number of interpolated channels (F(1,12)  = 0.48, n.s.). The remaining trials were sorted by picture category (HiPos, LoPos, Neut, LoNeg, HiNeg) and averaged within each category for every subject. After averaging, cortical generators of the ERFs were estimated based on the averaged response for each picture category using the L2-Minimum-Norm-Estimates method (L2-MNE) [43]. The L2-MNE is an inverse modeling technique that allows for estimating distributed neural network activity without a-priori assumptions regarding the location and/or number of current sources [44]. A sphere fitted to the scalp above a plane spanned by the nasion and both ear canals was used as conductivity model. A spherical shell with evenly distributed 2 (azimuthal and polar direction, radial dipoles do not generate magnetic fields outside of a sphere) x 350 dipoles was used as source model. A source shell radius of 87% of the individually fitted conductivity model was chosen, roughly corresponding to the grey matter depth. Although the distributed source reconstruction in MEG does not give the precise location of cerebral generators, it allows for a rather good approximation of cortical sources and corresponding assignment to larger cortical structures. Across all participants and conditions, a Tikhonov regularization parameter k of 0.1 was applied.

Topographies of source direction independent neural activities were calculated for each individual participant, picture category, and time-point. The resulting L2-MNE topographies of all individuals were used to identify differences in the processing of emotional information during the time-intervals and brain regions of interest. Two time-intervals of interest were determined a-priori based on previous findings regarding the processing of emotional stimuli. More specifically, we investigated differences in the processing of emotional information in an EPN-m (120–300 ms) and in an early (60–80 ms) time-interval, as both time-intervals show reliable processing differences between stimuli varying in hedonic valence and emotional arousal [30], [45]. During these time-intervals, we expected differential effects in ventral visual areas as well as the prefrontal cortex, following the results of previous ERF research investigating chronic PTSD [6], [28]. We hypothesized that ventral visual areas would reveal differences between the two groups during the EPN-m time-interval, the direction of which would be consistent with a decreased differentiation of emotional and neutral stimuli in the patient compared to the control group (i.e., avoidance). We further hypothesized that the prefrontal cortex, especially its bilateral orbitofrontal regions, would show group differences in the processing of emotional material during the early (60–80 ms) time-interval, the direction of which would indicate enhanced responses to emotional stimuli in the patient compared to the control group (i.e., hypervigilance).

Testing these hypotheses, we first calculated t-tests for the a priori defined arousal contrast (4, −1, −6, −1, 4) reflecting a linear arousal-driven neural activation pattern towards the respective category of the picture (HiPos > LoPos > Neut < LoNeg < HiNeg) across all patients and controls for the mean neural activations of each test source for both time intervals. Based on the cluster size of test sources with t-values exceeding a critical alpha level of p<0.05, clusters of six neighboring sources were defined as basis for non parametric testing as suggested by [46]. As part of this procedure, the above contrast analysis was calculated 1,000 times based on permuted drawings of data sets of all experimental conditions and subjects. Cluster-level statistics were then calculated by taking the sum of t-values of the above defined cluster size. Only clusters of summed t-values exceeding the random permutation cluster based alpha level of p<.05 were reported and then further analyzed with a second-level one-way ANOVA including the factor PICTURE CATEGORY (HiPos, LoPos, Neut, LoNeg, HiNeg) with a priori contrasts.

In a second step, we calculated t-tests for the mean activation of each test source during both time-intervals and looked for clusters of dipoles that would differentially express the a-priori contrast for the patient and the control group (4, −1, −6, −1, 4, −4, 1, 6, 1, −4). We then carried out the non parametric testing (specified above) on the basis of a cluster size of six neighboring dipoles and 1,000 permutations. Cluster-level statistics of clusters exceeding a cluster based alpha level of p<.05 were subjected to a second-level mixed-model ANOVA with the factors PICTURE CATEGORY (HiPos, LoPos, Neut, LoNeg, HiNeg) and GROUP (patients, controls). Post-hoc analyses were carried out to learn more about the nature of group differences and group by picture category interactions with regard to emotional picture processing.

This non parametric procedure was chosen to guarantee that the overall probability of a type-I error (i.e., of falsely rejecting the null hypothesis) was limited to 5%, although multiple comparisons (i.e., one t-test for every dipole within the a-priori defined time-interval) were calculated. Thus, all effects are significant on a non parametric level with p<.05. Nevertheless, the F- and p-values of the parametric test statistics (i.e., the one-way and the mixed-model ANOVAs) are also reported, because they specify the direction and the interpretation of the results and because they facilitate the comparison of our results with previous findings.

Although we especially targeted effects in the ventral visual and prefrontal cortex areas, we adjusted the a-priori defined source regions of interest on the results of the first-level analysis. We opted for this approach, because – in EEG and MEG – the estimation of neural activity at one specific location is not completely independent from simultaneous activations at other regions, at least as long the exact locations and time courses of all simultaneous activations are not completely known. This dependency is a direct consequence of the inverse problem of electrophysiology and it distinguishes inverse modelling in EEG/MEG from other neuroimaging methods with lower temporal resolution, such as fMRI or PET. An exact prediction of locations and time courses of difference activations between different experimental conditions, subject groups, or interactions is, thus, difficult and usually only possible if identical spatio-temporal source distributions are expected, such as in exact replication studies. A-priori predictions, which assume a specific pattern of group by valence interaction and which require this pattern to be significant for at least 20 ms in a predefined time interval and within at least 10 neighbouring sources of a predefined region, appear unrealistic, if the predictions are based on studies with different designs and subject groups.

Such an adjustment of the source regions of interest based on the first-level analysis may enhance the chance of type-I errors due to the high number of F-tests calculated in the first-level analysis. We, thus, minimized a probability of type-I errors by using spatio-temporal constraints, which were based on our previous experience with EEG/MEG studies, in which early affective processing had been investigated using the L2-Minimum-Norm method. However, as with all parametric tests, these constraints were not adjusted to the specific data distribution. The nonparametric testing procedure (as explained above) additionally guarded against the chance of residual type-1 errors and strengthened the validity of the present results.

### Subjective ratings of valence and arousal

After MEG-measurement, participants rated the hedonic valence and emotional arousal of 36 representative IAPS pictures (6 for each affective category and 12 neutral pictures, randomized order) using the Self Assessment Manikin (SAM) scales of valence and arousal [47]. Both scales ranged from 1 to 9, with low scores indicating negative or low-arousing pictures respectively.

## Results

### Psychological assessment

In patients, mean CAPS score was 81.57 (SD: 10.81, range: 68–94), mean IES score was 83.29 (SD: 7.41, range: 73–92), mean HADS score for anxiety was 15.43 (SD: 3.05, range: 11–20) and the mean score for depression was 12.57 (SD: 3.87, range: 8–17). All seven patients fulfilled all criteria of PTSD as specified in the CAPS (criteria A–D) except the time criterion E. Four patients fulfilled criteria of a comorbid major depression of moderate severity. No other comorbid diagnosis of axis I or II was given.

No significant correlation (spearman correlation) of the psychological assessments with the age of the patients (IES score r = 0.25, n.s.; HADS anxiety r = −0.63, n.s., HADS depression r = −0.31, n.s.) or the days since the trauma exposure (IES score r = 0.31, n.s.; HADS anxiety r = −0.49, n.s., HADS depression r = −0.02, n.s.) was detectable.

### Neural sources

**Figure 1:**
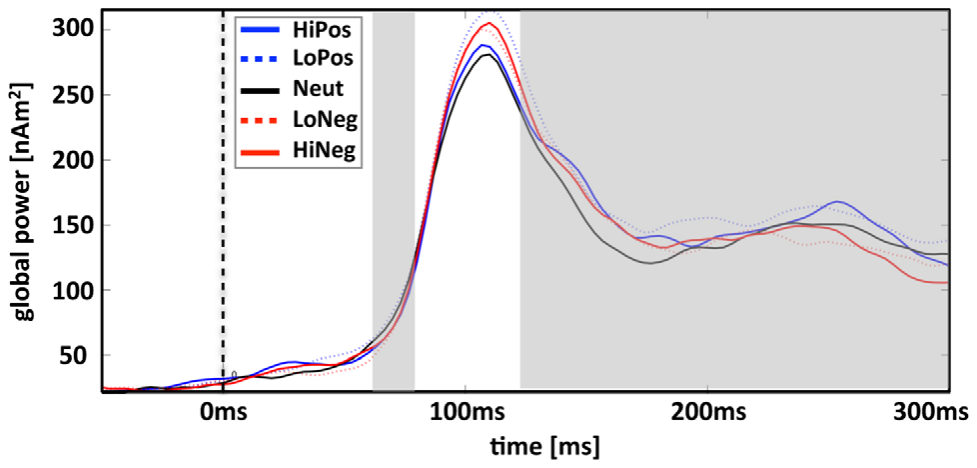
Plot displaying the global power of the estimated neural activity across time over all participants and all test sources. The two a-priori defined time-intervals of interest used for statistical analysis (60–80 ms, 120–300 ms) are marked in gray.

Analysis of global power plots revealed the expected emotional modulation of neural activation during the EPN-m (120–300 ms) and during a very early time-interval (<100 ms) (Figure 1; the global power is the sum of the squared estimated neural activities across all test sources within the source space calculated for each time point).

#### EPN-m time-interval (120–300 ms)

**Figure 2:**
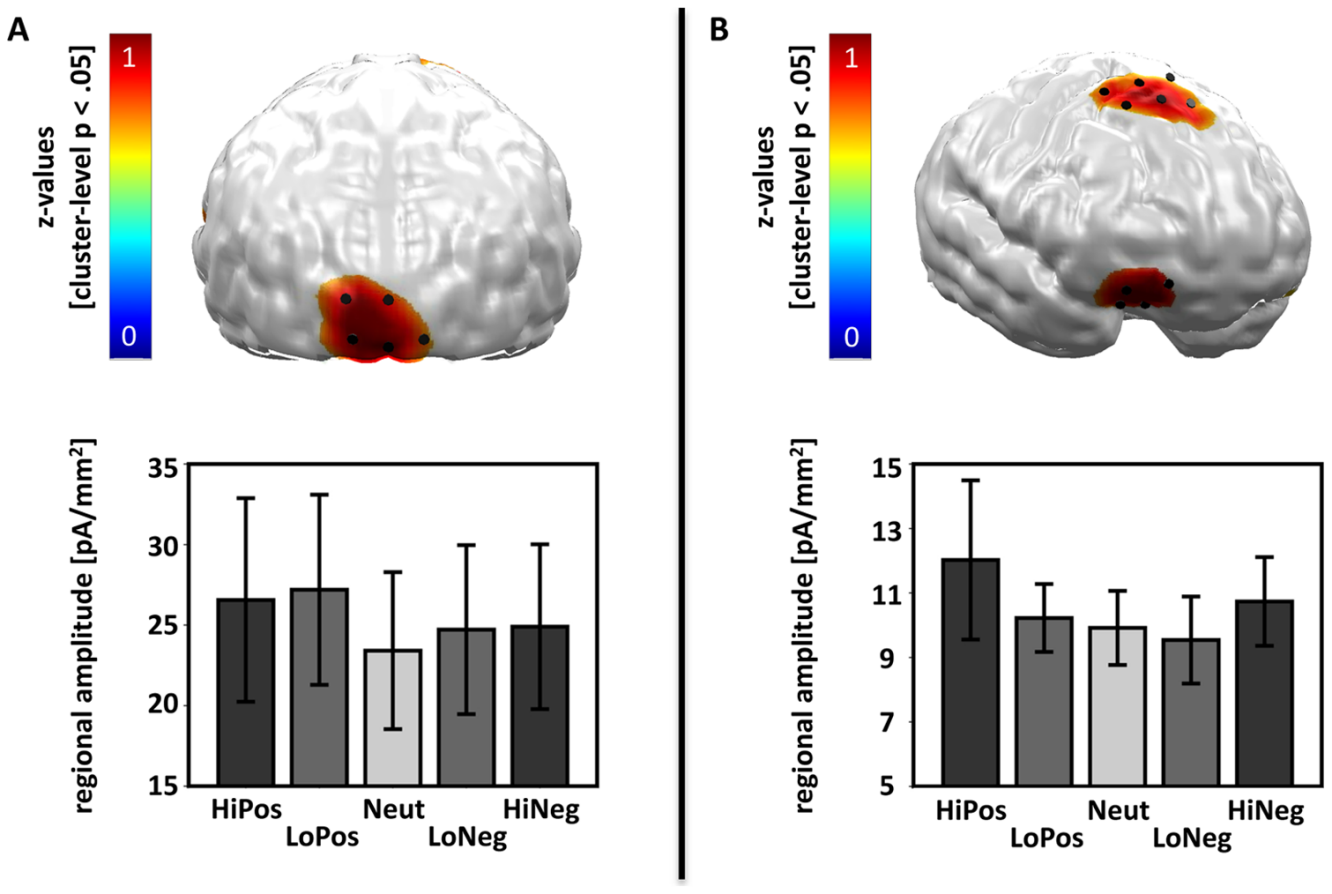
Visualization of the linear arousal-driven modulation of neural activation across all participants during the EPN-m time-interval (120–300 ms). A) In the top row of the first column, neural clusters are displayed that show a cluster-level significance of p<.05 for the linear arousal contrast calculated across all participants. The image was achieved by first calculating the contrast distribution (4, −1, −6, −1, 4) for the linear arousal contrast of PICTURE CATEGORY (HiPos > LoPos > Neut < LoNeg < HiNeg) across all patients and controls with averaged z-values of a cluster containing a center dipole and its five closest neighbours. The contrast distribution was then masked with the significance level topography for this very contrast after 1,000 random permutations of all data sets (subjects x conditions) given a significance criterion of p<.05 and a cluster size of six neighbouring dipoles. The masked contrast distribution, which highlights spatio-temporal clusters (6 dipoles, 120–300 ms) with significant effects on a cluster level, is displayed in z-values and projected onto a standard brain (back view). Reddish coloured areas indicate linearly increasing neural activity with increasing arousal of the picture categories visible at predominately medial occipital cortex areas. Black cylinders visualize the occipital dipole locations used for the second level analysis. Please mind that dipoles within these areas represent the centres of cluster-level significant groups of dipoles. The extent of the area with significant effects is thus bigger than the highlighted region. In the bottom row of the first column, the regional amplitude of the analyzed dipoles with regard to each picture category is displayed across patients and controls. B) as in A) with the exception that the masked contrast distribution projected on a standard brain is shown from the front and indicates a linearly increasing neural activity with increasing arousal of the picture categories at the dorsomedial and right orbitofrontal cortex.

The contrast distribution of z-values indicated a significant linear arousal-driven neural modulation across both groups in the medial occipital cortex (Figure 2A) and in the dorsomedial and right orbital prefrontal cortex (Figure 2B). The second-level one-way ANOVA with a-priori contrasts for the factor PICTURE CATEGORY calculated across the medial occipital cortex yielded a significant linear effect of emotional arousal (F(1,13)  = 5.02, p = .043), which indicated that for both patients and controls neural activation was greatest towards high-arousing pictures and smallest towards neutral pictures. The one-way ANOVA calculated across the dorsomedial and right orbital prefrontal cortex revealed a (by trend) significant linear arousal effect (F(1,13)  = 3.41, p = .088) that showed the same pattern as the convergent occipital effect across all participants.

**Figure 3:**
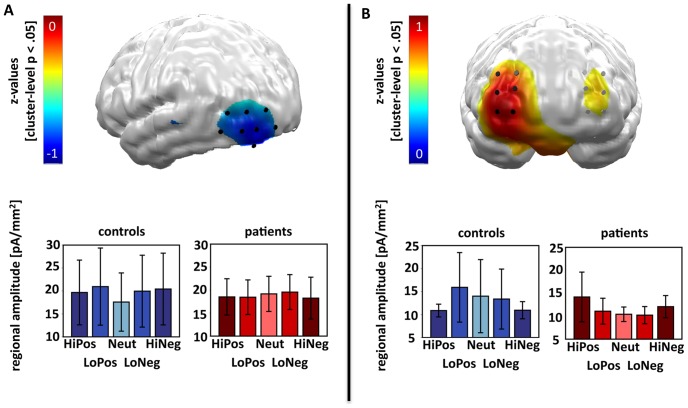
Visualization of differences between patients and controls with regard to the linear arousal-driven modulation of neural activation during the EPN-m time-interval (120–300 ms). A) In the top row of the first column, the neural clusters are displayed that show a cluster-level significance of p<.05 for the differences of patients and controls in the linear arousal contrast. The image was achieved by first calculating the contrast distribution (4, −1, −6, −1, 4, −4, 1, 6, 1, −4) for the interaction of the linear arousal contrast of PICTURE CATEGORY (HiPos > LoPos > Neut < LoNeg < HiNeg) with GROUP (patients, controls) with averaged z-values of a cluster containing a center dipole and its five closest neighbours. The contrast distribution was then masked with the significance level topography for this very contrast after 1,000 random permutations of all data sets (subjects x conditions) given a significance criterion of p<.05 and a cluster size of six. The masked contrast distribution, which highlights spatio-temporal clusters (6 dipoles, 120-300 ms) with significant effects on a cluster level, is displayed in z-values and projected onto a standard brain (back view). Bluish coloured areas indicate a decreased linear U-shaped arousal contrast for the picture categories in patients vs. controls visible at predominately left occipito-temporal areas. Black cylinders visualize the left occipito-temporal dipole locations used for the second level analysis. In the bottom row, the regional amplitude of the analyzed dipoles with regard to each picture category is displayed for patients and controls separately. B) as in A) with the exception that the masked contrast distribution projected on a standard brain is shown from the front and contains mainly reddish colours indicating an enhanced linear arousal modulation in patients vs. controls.

Furthermore, the contrast distribution of z-values indicated that patients and controls differed significantly with regard to a linear arousal effect in the left occipito-temporal (Figure 3A) and the bilateral orbitofrontal cortex (Figure 3B). A second-level mixed-model ANOVA with the factors PICTURE CATEGORY and GROUP calculated across the left occipito-temporal cluster supported this observation, by revealing a significant interaction of PICTURE CATEGORY and GROUP (F(1,12)  = 6.95, p = .022). The interaction was driven by a significant linear modulation of left occipito-temporal activation in controls (F(1,6)  = 6.00, p = .050) that was absent in patients (F(1,6)  = 1.45, n.s.). To further investigate the missing emotional modulation of neural responses in patients, we performed two additional analyses across the left occipito-temporal cluster, testing for an independent influence of picture AROUSAL (high-arousing, low-arousing pictures) and picture VALENCE (positive, negative pictures). The two-way ANOVA with the factors AROUSAL and GROUP failed to reveal an interaction of both factors (F(1,12)  = 0.08, n.s.), as did the ANOVA of VALENCE and GROUP (F(1,12)  = 0.54, n.s.).

With regard to the effects in the bilateral orbitofrontal cortex, the mixed-model ANOVA also indicated a (trend-level) significant interaction of PICTURE CATEGORY and GROUP (F(1,12)  = 3.46, p = .088). In contrast to the posterior activation, the prefrontal cluster showed a (by trend) linear arousal-driven modulation for the patient (F(1,6)  = 4.89, p = .069), but not for the control sample (F(1,6)  = 1.22, n.s.). We further explored the missing linear emotional modulation in the control sample, as we had done for the missing left occipito-temporal activation in patients. The ANOVA with the factors AROUSAL and GROUP revealed a (trend-level) significant interaction of AROUSAL and GROUP (F(1,12)  = 4.60, p = .053), but again only the patients showed enhanced processing of high-arousing compared to low-arousing pictures (t(6)  = 2.06, p = .043), while controls did not show any orbitofrontal cortex modulation by AROUSAL (t(6)  = −1.42, n.s.). An interaction of VALENCE and GROUP was not significant (F(1,12)  = 0.04, n.s.). In summary, in the EPN-m time interval the occipito-temporal and the orbitofrontal cortex showed diametrically opposed effects, with the first revealing enhanced responses to emotional stimuli in controls, but not patients, and the latter indicating enhanced response to emotional stimuli in patients, but not in controls.

Moreover, subjecting the left occipito-temporal and the right orbitofrontal neural clusters to a mixed-model ANOVA including the factors PICTURE CATEGORY, GROUP, and NEURAL CLUSTER (left occipito-temporal, right orbitofrontal), we found a significant interaction of all three factors (F(1,12)  = 7.38, p = .019). This finding supports the interpretation that patients and controls differentially recruit frontal or visual areas in the processing of emotional stimuli during the EPN-m. Patients and controls did not differ in the general recruitment of the two areas, as there was neither a significant main effect of PICTURE CATEGORY nor a significant main effect of GROUP for the left occipito-temporal (Fs(1,12)  = 1.10 and 0.08, n.s.) nor for the bilateral orbitofrontal cortex (Fs(1,12)  = 0.36 and 0.45, n.s.).

#### Early time-interval (60–80 ms)

**Figure 4:**
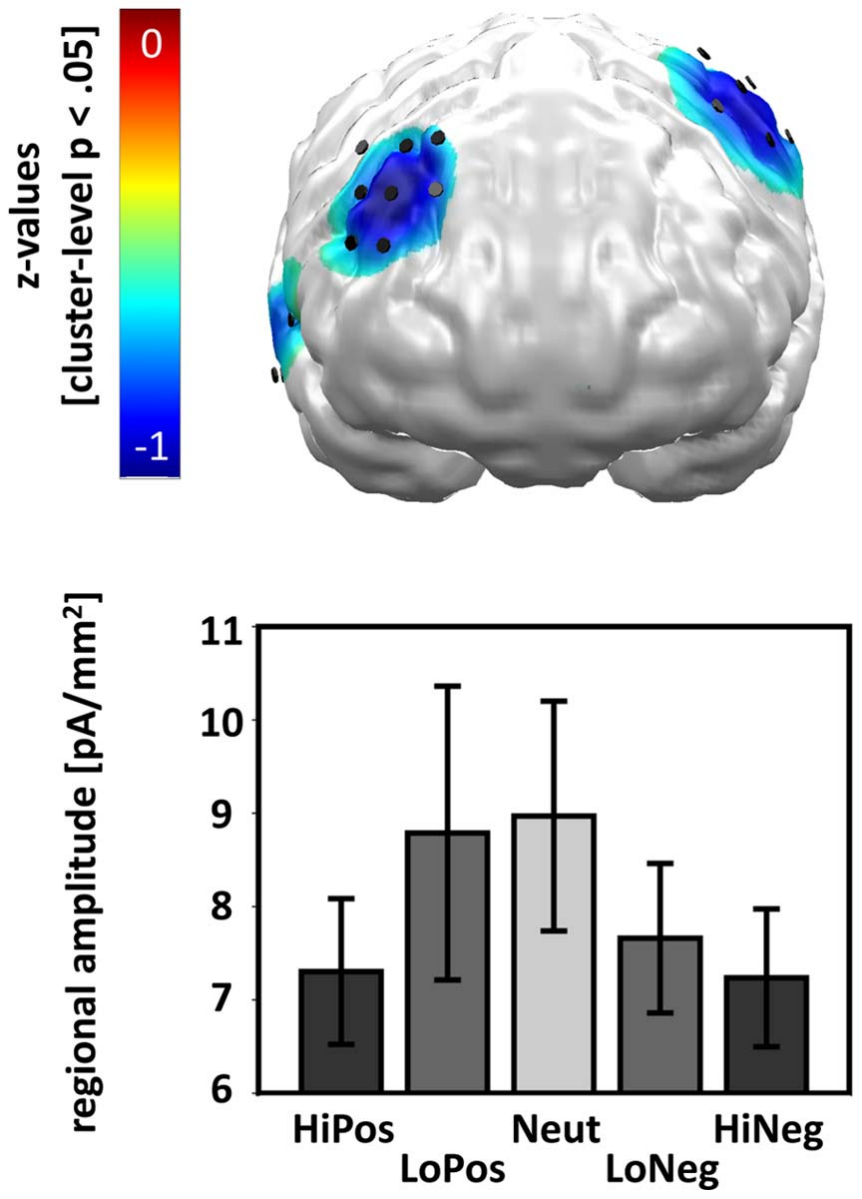
Visualization of the linear arousal-driven modulation of neural activation across all participants during the early time-interval (60–80 ms). A) as in 2A) with the exception that the contrast distribution is projected onto a standard brain shown from the front. It contains mainly bluish colours which indicates linearly decreasing neural activity with increasing arousal of the picture categories visible at predominately right dorsolateral and right and left temporal cortex.

The contrast distribution of z-values indicated a significant linear arousal-driven neural modulation in both patients and controls in the right dorsolateral and temporal as well as the left temporo-parietal cortex (Figure 4). The second-level one-way ANOVA with a-priori contrasts for the factor PICTURE CATEGORY calculated across both groups supported this observation with a significant arousal-driven effect of PICTURE CATEGORY (F(1,13)  = 14.17, p = .002). However, the direction of the linear arousal-driven effect was reversed, as both patients and controls revealed greatest neural responses towards neutral pictures and lowest neural responses towards high-arousing pictures.

**Figure 5:**
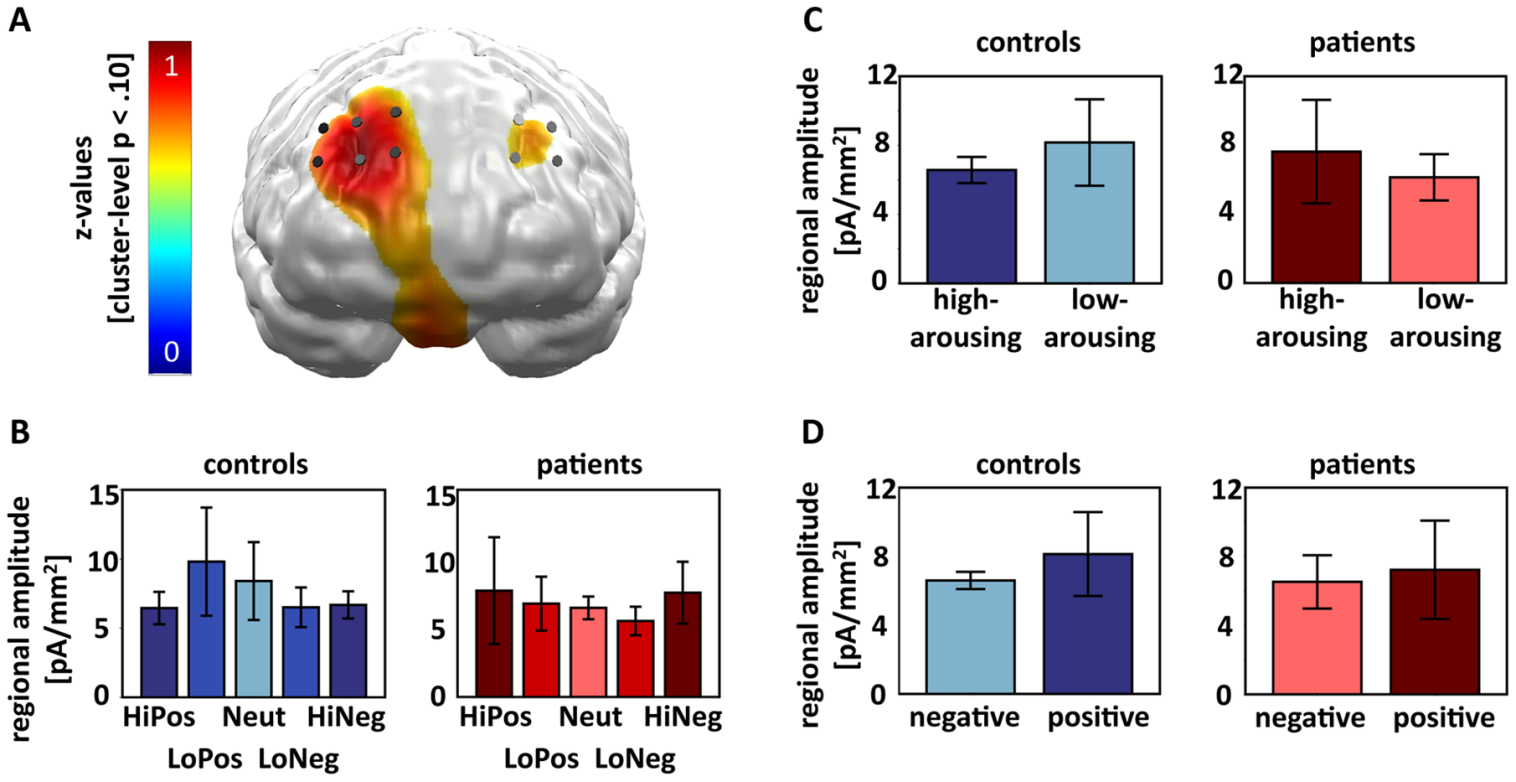
Visualization of differences between patients and controls with regard to the linear arousal-driven modulation of neural activation during the early time-interval (60–80 ms). 5A) and 5B) as in 3B) with the exception that a greater linear arousal modulation for patients compared to controls is found at predominately bilateral dorsolateral and ventro-central prefrontal dipole locations. Please note that the cluster-level significance has been increased to p<.10. 5C) The regional amplitude of the analyzed dipoles is displayed for patients and control separately with regard to the AROUSAL dimension of the picture (high-arousing, low-arousing picture) only. 5D) The regional amplitude of the analyzed dipoles is displayed for patients and control separately with regard to the VALENCE dimension of the picture (negative, positive picture) only.

Furthermore, the arousal by group contrast distribution of z-values revealed a significant differential linear arousal-driven activation pattern in the bilateral dorsolateral and the ventro-central prefrontal cortex (Figure 5A). The two-way ANOVA testing for a differential linear arousal modulation of PICTURE CATEGORY across GROUPS yielded a (trend-level) significant interaction of both factors (F(1,12)  = 4.62, p = .053), with main effects of PICTURE CATEGORY (F(1,12)  = 0.14, n.s.) and GROUP (F(1,12)  = 0.69, n.s.) not being significant. A separate analysis for patients and controls (Figure 5B) showed that patients did not show a linear arousal-driven modulation (F(1,6)  = 1.15, n.s.), while (by trend) controls did (F(1,6)  = 5.04, p = .066). The linear arousal-driven modulation in controls expressed greatest activation for neutral and low-arousing pictures and smallest activation for high-arousing pictures. To further investigate the missing linear arousal-driven modulation in patients, we performed additional analyses across the dorsolateral-ventro-central cluster, testing for independent effects of picture AROUSAL (Figure 5C) and picture VALENCE (Figure 5D). A two-way ANOVA with the factors AROUSAL and GROUP indicated a differential effect in the arousal modulation of prefrontal brain activation (F(1,12)  = 6.26, p = .028). One-sided t-tests indicated greater activation for high-arousing than low-arousing pictures in patients (t(6)  = 2.02, p = .045) and (by trend) greater responses towards low-arousing than high-arousing pictures in controls (t(6)  = −1.61, p = .079). The two-way ANOVA of VALENCE and GROUP revealed a (trend-level) significant main effect of VALENCE, only, (F(1,12)  = 4.12, p = .065), which stemmed from relatively greater processing of positive than negative pictures in controls (t(6)  = 1.76, p = .065) and a lack of valence differentiation in patients (t(6)  = 1.05, n.s.). Main effects of GROUP were not significant, neither for the AROUSAL x GROUP (F(1,12)  = 0.21, n.s.) nor for the VALENCE x GROUP (F(1,12)  = 0.21, n.s.) ANOVA.

Summing up the effects in the early time-interval, we found a linear arousal-driven neural activation in the right dorsolateral and temporal cortex as well as in the left temporo-parietal cortex that is consistent with enhanced activation for neutral and low-arousing compared to high-arousing pictures in both patients and controls. The bilateral dorsolateral and ventro-central prefrontal cortex areas were differentially modulated depending on the group. While patients showed enhanced responses towards high-arousing pictures, controls showed relatively increased activation for low-arousing and positive pictures.

### Valence and arousal ratings

**Figure 6:**
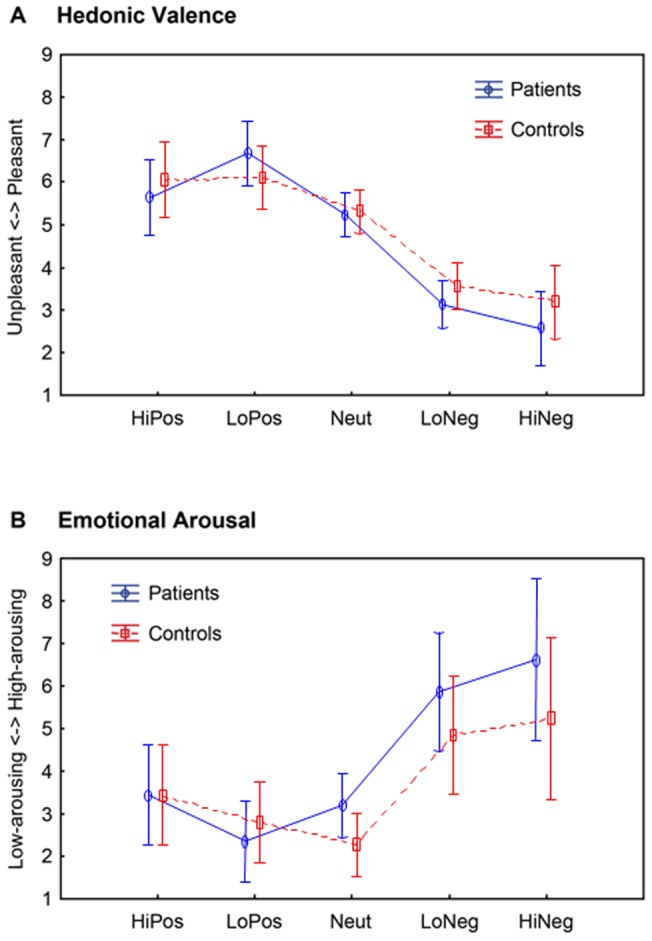
Visualization of subjective ratings of (A) hedonic valence and (B) emotional arousal across all patients (blue) and controls (red).

SAM-ratings of valence (Figure 6A) and arousal (Figure 6B) were analysed using mixed-design ANOVAs with the factors PICTURE CATEGORY and GROUP. Both groups distinguished between picture categories, as indicated by differential valence (F(4,48)  = 44.72, p<.001) and arousal ratings (F(4,48)  = 33.41, p<.001) (Figure 6). However, patients and controls did not differ in their valence (F(4,48)  = 1.04, n.s.) or their arousal ratings (F(4,48)  = 2.08, n.s.).

## Discussion

In the present study, we compared acutely traumatized PTSD patients to healthy controls with regard to emotion processing in an EPN-m (120–300 ms) and in an early (60–80 ms) time-interval and investigated whether the hypervigilance-avoidance reaction seen in chronic PTSD patients would already be present immediately after trauma. Across both patients and controls, we observed a linear U-shaped arousal modulation of the occipital cortex and the dorsomedial and right orbital prefrontal cortices during the EPN-m and a linear, but inverted arousal modulation of the right and left temporal and right dorsolateral prefrontal cortices during the early time-interval. We report that acutely traumatized PTSD patients show an exaggerated response towards arousing stimuli in the bilateral orbitofrontal cortex and a reduced affective differentiability in occipito-temporal areas during the EPN-m time-interval. Furthermore, acutely traumatized PTSD patients reveal exaggerated prefrontal activation towards high-arousing stimuli already during very early stages of visual processing (i.e., 60 to 80 ms), during which prefrontal cortex areas in controls preferentially process low-arousing and positive pictures.

During the early and the EPN-m time-interval, we observed a linear arousal-driven modulation present across patients and controls that is somewhat consistent with previous results of ERP/ERF research in healthy subjects. The inverted linear arousal effect in the left parieto-temporal cortex during the early time-interval matches the results of two conditioning studies which observed enhanced responses towards approach-related visual [48] and auditory [49] information in the left temporo-parietal junction during 50 to 80 ms and during a N1 m time-interval, respectively. In addition, it could be speculated that the inverted linear arousal effect in the right dorsolateral prefrontal cortex during the early time-interval may be related to an inhibitory role of this area on subsequent emotion processing, as it has been implicated in emotion regulation [50]. Moreover, the U-shaped linear arousal effect observed during primary visual areas and orbitofrontal and dorsomedial cortices fits to studies that argue for a great role of these areas in an extended emotion processing network which is typically active during an EPN-m time-interval [51], [52]. Thus, the existence of common neural emotion processing across patients and controls may suggest that despite the trauma acute PTSD patients preserve part of the emotion network and its function as it was before trauma exposure. However, acute PTSD patients also show a variety of neural responses that are exaggerated or inhibited in comparison to healthy controls. We will discuss the most prominent of these responses in the next paragraphs.

The enhanced activation of prefrontal cortex regions towards arousing stimuli, which we found in patients during an early and an EPN-m time-interval, is consistent with results of previous neuroimaging studies investigating acutely traumatized subjects. In a positron emission tomography (PET) study, recent motor vehicle collision survivors revealed greater resting-state activation in PFC areas compared to healthy controls [32]. Similarly, enhanced PFC activation in acutely traumatized earthquake survivors was present less than 4 weeks after trauma using resting-state functional magnetic resonance imaging (fMRI) [33].

The pattern of an exaggerated early PFC response followed by a reduction in affective differentiability within posterior regions during the EPN-m time-interval matches the alterations found in chronic PTSD patients. Indeed, in several PET studies, chronic PTSD patients reveal increased OFC activation during symptom provocation [14], [53] and auditory task performance [54]. ERP/ERF studies indicate enhanced PFC activation in chronic PTSD during the first 100 ms after stimulus onset [6], [55] and reduced emotional differentiation in posterior sensory regions [6]; [27]; [28]; [56]. These converging results support the conclusion that these specific changes in brain function observed in chronic PTSD patients are not necessarily due to adaptation processes caused by years of suffering from a mental disorder, but can arise immediately as a result of severe trauma.

The reported findings are consistent with the idea that severe trauma leads to immediate, enduring changes in brain function and to the genesis of a trauma network [2], [7]. Such a trauma network may function as a rapid threat detection mechanism, which triggers a defensive reaction, although conscious stimulus appraisal might not have yet occurred [57]. The brain might make use of such a mechanism in situations of anticipated danger, cutting back on detailed stimulus analysis and shifting neural activity from sensory cortices to cortical action-related areas [6]. With regard to the present study, this mechanism may correspond to the activation of prefrontal cortex areas during early stimulus processing. A number of pre-clinical studies support this consideration by reporting prefrontal, in particular orbitofrontal, activation in emotional learning and categorization. For example, the PFC has been associated with stimulus reinforcement learning in macaques [58], [59] and in humans [60], [61]. A recent olfactory conditioning study revealed that early (50–80 ms) and EPN-m (130–190 ms) prefrontal activation differentiated between aversively conditioned and unpaired faces, although participants lacked contingency awareness [52]. The authors assume the PFC to be involved in rapid stimulus classification independent of conscious awareness or detailed stimulus analysis. Of interest, several candidate pathways, such as a thalamo-amygdaloid or a geniculo-cortico-cortical pathway, have been proposed which could mediate such rapid prefrontal activations (cf. 52). Taken together, reviewed findings support the existence of a rapid prefrontal threat detection mechanism in PTSD which – as the results of the present study suggest – might be especially sensitive to the arousal, but less so to the valence dimension of a stimulus.

Although enhanced PFC responses are supported by several fMRI and PET studies, there also are contradictory reports of a prefrontal hypoactivation in PTSD [15]. More specifically, PTSD patients show reduced prefrontal activation during symptom provocation [62], [63], retrieval of fear-related words [13], or verbal memory encoding [64]. Differences in the direction of PFC responses might depend on the duration of PTSD, i.e. on the phase of the mental disorder. Indeed, PET-studies investigating acutely traumatized participants show a hyperactivation of PFC areas [32], [33], while hypoactivation is recorded in patients with childhood traumatization [13], [64]. Moreover, in chronic PTSD, PFC hypoactivation shows an inverse functional relationship to enhanced amygdala activation, while PFC and amygdala responsiveness are positively correlated in rather acutely traumatized subjects [65]. The apparent contradiction of a PFC hyperactivation in acute PTSD patients during early and EPN-m processing stages as found here in MEG with a prefrontal hemodynamic hypoactivation in response to aversive stimuli in PTSD patients might also reflect the differential temporal sensitivity of electrophysiological and hemodynamic measures: the PFC effects were measurable for roughly a fiftieth of a second (60–80 ms) during the early time-interval and around a sixth of a second (120–300 ms) during the EPN-m time-interval. Such highly transient effects may not necessarily lead to measurable hemodynamic changes as BOLD changes reflect the superposition of neural activity across several seconds. In fact, it could well be supposed that prefrontal BOLD hypoactivations mainly reflect a long lasting down regulation of the early and transient hyperactivations which may act as a kind of compensation strategy and as part of the subsequent avoidance reaction. This interpretation is of course quite speculative and awaits further confirmation. It is also worth noting in this respect that source localization in MEG depends on several assumptions and is by far not as spatially accurate as is fMRI. Indeed, MEG source reconstruction always involves a spatial uncertainty.

Interestingly, controls show (by trend) increased activation for positive compared to negative and for low-arousing compared to high-arousing stimulus material in the PFC during 60–80 ms. The first finding differs from results of previous PTSD studies, in which healthy controls did not reveal any early prefrontal differentiation between positive, negative, and neutral [28] or negative and neutral stimuli [6]. Such divergent results may depend on age-related differences between study samples, since the healthy control sample used here was considerably older (mean: 43.5 years) compared to previous studies (mean: 26 years) [6], [28]. Indeed, there is a great body of literature reporting differences in the processing of positive and negative information between young and mature adults. Studies reveal a linear negative trend with regard to the processing of negative material and aging, that is neural responses towards negative stimuli decrease with advancing age, while processing of positive and neutral material remains constant [66], [67]. Importantly, such age-related effects may be associated with differences in PFC activation. Young adults show enhanced responses in the ventromedial PFC (vmPFC) towards negative compared to positive material, while the neuronal activation pattern is reversed in older adults [68]. Facilitated processing of positive material in the vmPFC could result from a greater cognitive control over negative stimuli, as indicated by a greater activation of the medial PFC (mPFC) towards negative material in older adults [69]. Thus, the greater activation towards positive material found here in healthy controls can be explained by a positivity bias that comes with age. Apart from this ‘positivity bias’, healthy controls also show (by trend) greater PFC activation for low-arousing than for high-arousing material. It could be assumed that this finding relates to a greater cognitive control not only over negative, but also over high-arousing material in mature adults. As proposed in the socioemotional selectivity theory [70], emotion regulation may become a more important goal with advancing age, which could result in greater cognitive control over negative and highly arousing material and, thus, decreased neural responses for both stimulus categories. However, future research should investigate how age modulates the neural processing of stimuli varying in emotional arousal.

Following the prefrontal hyperactivation, acutely traumatized patients reveal a reduced affective differentiability within the left occipito-temporal cortex region during the EPN-m. Such a suppression of emotion-specific EPN-m activation is consistent with previous electrophysiological studies, reporting a reduction of occipital discrimination with regard to emotional faces, words, and pictures [20], [28], [71] in PTSD patients and subjects with early life stress [56] in a similar time-interval. Typically, reduced brain responses are associated with inhibited processing [72], which suggests that the EPN-m suppression indicates reduced affective processing of emotional pictures in sensory cortices. Furthermore, it has been proposed that such inhibited processing could reflect a gating mechanism [20], [71], which controls for possibly threatening perceptual input and restricts such information from being further processed. Several studies provide support for this hypothesis by reporting a gradual decrease in brain activation to tones of increasing intensity in PTSD [73], [74]. However, there is also an alternative explanation for the EPN-m specific inhibition. In accordance with the hypothesis of emotional attention [75], enhanced responses have been associated with increased attention, while lower brain activation seems to signal reduced emotional attention. Thus, all stimuli seem to receive less emotional attention in ventral visual areas during the EPN-m in PTSD. This decrease of attention could be caused by enhanced PFC activation during earlier processing stages. Since the rapid alarm system has already distinguished between threatening and neutral pictures, visual attention in sensory cortices ceases from being necessary (cf. 28). 

Visual emotion processing in acute PTSD patients overlaps with emotion processing in chronic PTSD patients, apart from one finding: Acute PTSD patients did not only show an enhanced PFC response to arousing stimuli during the early, but also during the EPN-m time-interval. Such a sustained prefrontal response to potentially threatening stimuli corresponds to the PET and fMRI investigations of acute PTSD patients [32], [33], mentioned earlier. In context with the proposed positive correlation of prefrontal and amygdala activity in acute, but not in chronic PTSD patients [65], the sustained PFC activation could indicate a greater need of PFC-driven down-modulation on excitatory cortical or sub-cortical areas, such as the amygdala. In line with such an inhibitory PFC modulation, it has been proposed that enhanced PFC and decreased amygdala activation after trauma are indices of a positive prognosis [32]. In contrast, assuming a mechanism of emotional attention [75], the PFC activation during the EPN-m could also reflect that the hypervigilant response towards threatening stimuli during the early time-interval is carried over towards later processing stages. Thus, we speculate that not only early, but also later stages of visual processing, which are associated with a more detailed stimulus analysis, are overshadowed by the need of trauma survivors to rapidly detect threatening events. Moreover, late, but not early PFC responses towards threat-related stimuli have been shown to be subject to extinction [76], which may explain the finding of enhanced prefrontal EPN-m activation in acute, but not chronic PTSD patients. However, with the present data available, this matter cannot be satisfactorily settled. We thus stress the need for ERP/ERF studies on the long-term development of acute trauma survivors.

At visual inspection, acute PTSD patients seem to report enhanced arousal and reduced valence ratings for unpleasant pictures (Figure 6), which converges with the electrophysiological findings. However, these differences could not be verified statistically.

The findings of the present study have to be interpreted cautiously with respect to certain limitations. The rather small number of patients that could be investigated appears as main limitation. This constrains the degree to which results can be generalized to the population of acutely traumatized patients, i.e. it does not answer the question which percentage of traumatized patients may develop an H-A reaction. Nevertheless, it does not compromise our positive test result of the specific hypothesis that neural hypervigilance-avoidance reactions can arise as an almost immediate result of acute PTSD. In fact, the very distinct hypothesis with an early prefrontal hyperactivation followed by a sensory hypoactivation in acute PTSD compared to controls was carried out in close accordance to previous studies in chronic PTSD patients. The clear a priori definitions of regions and time-intervals of interest as well as effect directions within each region and time-interval speaks for a very low probability of false positive results due to a limited sample size. The non parametric approach further strengthens the informative value of our findings.

The investigated patients already revealed a high comorbidity with depression, which suggests that the effects cannot be solely attributed to PTSD. Furthermore, recent studies show effects of reduced affective modulation in temporo-parietal areas [77] and during the EPN-m [56] in depression similar to PTSD. Thus, the present findings may reflect correlates of both a post-traumatic stress as well as a post-traumatic depressive disorder. To explore to what degree the present results could be thought of as being independent of a depressive symptomatology in the investigated patients, we included depressive symptomatology (HADS depression sub-score) as a covariate into the analysis. Inconsistent with [28], the effects ceased to be significant, which may be due to the high correlation of depressive and anxious symptomatology. Thus, we cannot assume that the results reflect correlates of PTSD that are independent of a depressive symptomatology. Future studies should investigate, whether the hypervigilance-avoidance pattern is associated with PTSD, depression, or a general traumatic stress reaction [78]. Finally, as an additional trauma-exposed control group without PTSD has not been investigated, we cannot exclude the alternative that trauma exposure alone even in absence of an acute stress reaction might suffice generating a hypervigilance-avoidance reaction.

Nevertheless, in spite of this limitations, our findings indicate that the early hypervigilance and the following avoidance pattern seen in chronic PTSD patients is not necessarily a product of a long endured mental disorder, but arises as an almost immediate result of severe traumatisation. Thus, traumatic life experiences can influence emotion processing strongly, leading to long-lasting changes in trauma network activation and expediting a chronic manifestation of maladaptive cognitive and behavioral symptoms. Future studies could try to investigate which factors promote and which prevent such immediate changes in trauma network activation. Information about individual vulnerability and protection could help to provide customized behavioral trainings in prevention and intervention. In this context, we would like to stress the importance of ERP/ERF studies investigating the processing of emotional stimuli. Although ERP/ERF studies cannot localize effects as well as can be done in fMRI and PET based research, ERP/ERF studies provide several advantages as compared to hemodynamic measures. The most important advantage of studies using EEG or MEG can be seen in the fact that EEG/MEG signals show the underlying neuronal processes much better than hemodynamic measures, as EEG/MEG signals directly reflect on-going neural activity. The temporal acuity of ERP/ERF data allows for identifying different stages of emotion processing and the disturbance thereof, which greatly enhances our understanding of the human brain. A comprehensive model of disturbed emotion processing in anxiety disorders, such as PTSD, should not only include the localization, but also the timing, and the course of effects, which is why future studies should increasingly make use of EEG or MEG or simultaneous EEG-MEG measurements.

## Supporting Information

Supporting Information S1 (PDF)

Supporting Information S2 (PDF)
