# Supplementary material for: Early Affective Processing in Patients with Acute Posttraumatic Stress Disorder: Magnetoencephalographic Correlates
Source: PLoS One. 2013 Aug 19;8(8):e71289. doi: 10.1371/journal.pone.0071289 (PMC3747150; doi:10.1371/journal.pone.0071289)
Supplement: Supporting Information S1 — (PDF) [file pone.0071289.s001.pdf]

**Anhang 3: Merkblatt zum Aufklärungsgespräch für Patienten über den Gesamt-Studienablauf.**

Westfälische
Wilhelms-Universität
Münster

Universitätsklinikum
Münster

**Institut für klinische Radiologie**

**Institut für Biomagnetismus und Biosignalanalyse**

**Klinik und Poliklinik für Psychosomatik und Psychotherapie**

Albert-Schweitzer-Str. 33
48149 Münster
Durchwahl: (02 51) 83 – 5 29 05

## Merkblatt zum Aufklärungsgespräch über Hirnuntersuchung mittels Magnetoenzephalographie (MEG) und funktioneller Magnetresonanztomographie (fMRT)

Liebe(r) Patient(in),

sie leiden unter einer posttraumatischen Belastungsstörung (PTBS). Die Post-traumatische ist eine psychische Störung, die als Reaktion auf ein traumatisches oder stark belastendes Ereignis auftritt, das die individuellen Möglichkeiten der betroffenen Person, mit diesem Trauma umzugehen, überfordert. Die PTBS ist nur eine der möglichen Folgereaktionen auf z.B. sexuellen Missbrauch, Vergewaltigung, Krieg, Katastrophen aller Art sowie der Diagnose einer bedrohlichen Krankheit. Charakteristisch für die PTBS sind Albträume, Schlafstörungen sowie das immer wiederkehrende unwillkürliche Nacherleben einer bedrohlichen traumatisierenden Situation in so genannten Flashbacks. Im Unterschied zur akuten Belastungsreaktion (Dauer der Symptome bis einen Monat) spricht man von PTBS ab einer Dauer von einem Monat.
Wie oben angemerkt ist das unwillkürliche Nacherleben einer bedrohlichen traumatisierenden Situation ein typisches Symptom der PTSD, auch wenn Sie dieses Symptom persönlich nicht unbedingt aufweisen müssen.
Obwohl diese Flashbacks unwillkürliche aufzutreten scheinen, können sie jedoch in manchen Fällen auch durch Reize (Geräusche, Gerüche, Bilder) hervorgerufen werden, welche Ähnlichkeiten mit Reizen aufweisen, die Sie während einer traumatischen Situation wahrgenommen haben. Dieses Hervorrufen bestimmter Erinnerungen durch bestimmte Reize schützt uns im Alltagsleben davor, ein bestimmtes Verhalten (z.B. streicheln eines knurrenden Hundes) auszuführen, welches bereits einmal in unserem Leben zu körperlichem oder seelischem Schmerz geführt hat - Knurren (Geräusch) und Zähnefletschen (Bild) führt zum Hundbiss (Schmerz). Dieser sehr sinnvolle und zum Teil überlebenswichtige Mechanismus kann leider jedoch auch die oben angemerkten sehr negativen Konsequenzen haben. Allerdings müssen solche Reize nicht zwangsläufig zu Flashbacks führen. Die auch unbewusste Wahrnehmung dieser Reize kann Sie auch nur unbemerkt von

einer zu bewältigenden Aufgabe ablenken und damit zu starken Aufmerksamkeitsstörungen führen.
Um die Ursachen und Konsequenzen einer Posttraumatischen Belastungsstörung besser verstehen zu können, möchten wir bei Ihnen untersuchen, wie die Durchführung einer schwierigen Aufmerksamkeitsaufgabe durch emotionale Hintergrundgeräusche gestört wird. Durch den damit erhofften Erkenntnisgewinn könnten zukünftige Behandlungen verbessert oder Erfolge von Therapien besser eingeschätzt werden. Sie dürfen durch diese Untersuchung also keinen direkten Einfluss auf Ihre Beschwerden erwarten, d.h. ein unmittelbarer therapeutischer Nutzen ist nicht vorhanden. Wir möchten Sie aber trotzdem bitten, an der Untersuchung teilzunehmen, in der wir mit Hilfe eines Magnetenzephalographen sowie eines Magnetresonanztomographen die Antwort des Gehirns während der Ausführung schwieriger Aufmerksamkeitsaufgabe vor dem Hintergrund emotionale Hintergrundgeräusche untersuchen. Dies wollen wir bei Patienten aber auch bei gesunden Menschen untersuchen. Dafür benötigen wir ihre Hilfe.

In den letzten Jahren ist es möglich geworden, die Aktivierung des Gehirns gefahrlos und ohne größere Belastung mit Hilfe der so genannten Magnetenzephalographie (MEG) sowie der funktionellen Magnetresonanztomographie (fMRT) bildlich darzustellen. Dabei macht man sich den Umstand zu Nutze, dass aktive Hirnregionen magnetische Felder erzeugen, die außerhalb des Kopfes mit dem MEG gemessen werden können. Darüber hinaus verbrauchen aktive Hirnregionen mehr Sauerstoff als nicht aktive und diese Unterschiede können durch fMRT aufgelöst werden.
Das fMRT liefert Ergebnisse mit hervorragender räumlicher Auflösung, allerdings kann es keine Aussagen über die zeitliche Abfolge von verschiedenen Hirnaktivierungen machen. Genau diese wichtigen Informationen liefert das MEG.
Im Rahmen dieser Studie werden Sie in Abhängigkeit der Zwischenergebnisse an einem der beiden oder an beiden Untersuchungen teilnehmen.
Aus diesem Grunde möchten wir Sie bitten, die beiden anliegenden Merkblätter zum Aufklärungsgespräch über die fMRT sowie MEG Untersuchung, in denen beide Verfahren und die geplanten Untersuchungen detailliert beschrieben werden, aufmerksam zu lesen.
Zusätzlich zu einem der beiden oder beiden Untersuchungen werden wir Sie noch bitten fünf Fragebögen, die uns wichtige Zusatzinformationen für die Datenauswertung liefern, auszufüllen.
Dabei handelt es sich
1) um einen standardisierter Fragebogen zur Erhebung des Ausmaßes psychischer Belastungen nach einem traumatischen Lebensereignis.
2) um einem standardisierter Fragebogen zur Erfassung von Symptomen einer PTBS
3) um einem Fragebogen zur Händigkeit, ob Sie also vornehmlich Rechts- oder Linkshänder sind.
4) um einem Fragebogen zur Überprüfung depressiver oder ängstlicher Symptome eines Probanden.
5) um einem standardisiertes klinisches Interview zur Erhebung verschiedener psychischer oder psychiatrischer Krankheitssymptome oder Störungen.

Mit Ihrer Unterschrift unter dieses Dokument erklären Sie die grundsätzliche Bereitschaft diese Fragebögen auszufüllen sowie an beiden Untersuchungen teil zu nehmen. Sie werden jedoch vor der jeweiligen MEG oder fMRT Untersuchung noch einmal über die Studie aufgeklärt und werden dann noch einmal gebeten einen Einverständnisbogen zur Teilnahme an der entsprechenden MEG oder fMRT

Untersuchung zu unterzeichnen. Sie können eine ursprüngliche Zustimmung aber auch jederzeit und ohne Angabe von Gründen wieder rückgängig machen, ohne dass sich Nachteile für Sie ergeben. Sie können auch eine laufende Untersuchung ohne Angabe von Gründen unterbrechen, ohne dass sich Nachteile für Sie ergeben.

Für die Teilnahme an den Untersuchungen entstehen Ihnen keine Kosten. Sollten Ihnen Fahrtkosten entstehen, werden diese bis zu einem Betrag von 20 Euro pro Untersuchung gegen Vorlage einer Rechnung erstattet. Fahrtkosten werden auch dann erstattet, wenn Sie eine Untersuchung auf eigenen Wunsch abbrechen.

Bitte beachten Sie:
Falls Sie sich entscheiden, an dieser Studie teilzunehmen, bitten wir Sie, uns schriftlich auf diesem Bogen ihr Einverständnis mitzuteilen. Sie können natürlich eine solche Zustimmung jederzeit und ohne Angabe von Gründen wieder rückgängig machen, ohne dass sich Nachteile für Sie ergeben.
Ihre Daten werden nur zum Zweck der Forschung ausgewertet. Ihr Name, Ihre persönlichen Daten, Ihre Angaben in den fünf Fragebögen und die Ergebnisse der MEG und/oder fMRT Untersuchungen unterliegen dabei selbstverständlich der ärztlichen Schweigepflicht.

Wenn Sie noch Fragen haben....
Bitte scheuen Sie sich nicht, weitere Fragen, die Sie möglicherweise im Zusammenhang mit dieser Untersuchung haben, dem Sie untersuchenden Arzt oder anderen Personen unserer Abteilung zu stellen. Sie können sich Ihre Entscheidung gerne in Ruhe überlegen. Sollten Sie zustimmen, bitten wir Sie, die folgende Einverständniserklärung zu unterschreiben.

**Einwilligungserklärung**

Name der Patientin / des Patienten____________________________

Ich bin über die geplante Studie eingehend und ausreichend unterrichtet worden. Ich konnte Fragen stellen, die Informationen habe ich inhaltlich verstanden. Ich habe alle Fragen des Probandenfragebogens wahrheitsgemäß beantwortet. Ich habe keine weiteren Fragen, fühle mich ausreichend informiert und willige hiermit nach ausreichender Bedenkzeit in die Untersuchung ein. Mir ist bekannt, dass ich meine Einwilligung jederzeit ohne Angaben von Gründen widerrufen kann. Ich weiß, dass die Untersuchung wissenschaftlichen Zwecken dient und die gewonnenen Daten eventuell für wissenschaftliche Veröffentlichungen verwendet werden. Hiermit bin ich einverstanden, wenn dies in einer Form erfolgt, die eine Zuordnung zu meiner Person ausschließt. Auch diese Einwilligung kann ich jederzeit widerrufen.

Münster,______________ ____________________________________
Ort, Datum Unterschrift der Patientin / des Patienten

| | |
|---|---|
| Münster,_______________ | _____________________________________ |
| Ort, Datum | Unterschrift des Klinikmitarbeiters |
