# Supplementary material for: Early Affective Processing in Patients with Acute Posttraumatic Stress Disorder: Magnetoencephalographic Correlates
Source: PLoS One. 2013 Aug 19;8(8):e71289. doi: 10.1371/journal.pone.0071289 (PMC3747150; doi:10.1371/journal.pone.0071289)
Supplement: Supporting Information S2 — (PDF) [file pone.0071289.s002.pdf]

Merkblatt zum Aufklärungsgespräch über die MEG Untersuchung.

Westfälische
Wilhelms-Universität
Münster

UKM
Universitätsklinikum
Münster

Institut für Biomagnetismus und Biosignalanalyse

Klinik und Poliklinik für Psychosomatik und Psychotherapie

Albert-Schweitzer-Str. 33
48149 Münster
Durchwahl: (02 51) 83 – 5 29 05

Hirnuntersuchung mittels Magnetoenzephalographie (MEG)

Liebe(r) Proband(in),

wir möchten Sie bitten, an einer Untersuchung teilzunehmen, in der wir mit der Magnetoenzephalographie (MEG) die Aktivität Ihres Gehirns bei der Wahrnehmung von emotionalen und neutralen Bildern untersuchen möchten. Die Untersuchungsergebnisse sollen dem besseren Verständnis dienen, wie unser Gehirn solche Reize verarbeitet. Dies wollen wir zunächst bei gesunden Personen, mittelfristig aber auch bei Patienten untersuchen, die unter Störungen der emotionalen Reizverarbeitung leiden. Wir erhoffen uns hierbei neue Erkenntnisse zur besseren Behandlung dieser Krankheiten. Dafür benötigen wir ihre Hilfe.

In den letzten Jahren ist es möglich geworden, die Aktivierung des Gehirnes gefahrlos und ohne jegliche Belastung mit Hilfe der so genannten Magnetenzephalographie (MEG) bildlich darzustellen. Während Sie eine geistige Aufgabe bewältigen, einen Sinnesreiz wahrnehmen oder eine Bewegung durchführen, wird die Arbeit Ihrer Nervenzellen im Gehirn elektrische und magnetische Felder generieren, die außerhalb Ihres Kopfes messbar sind. Diese werden mittels eines Magnetoenzephalogramms (MEG) durch eine Vielzahl von kleinen Sensoren erfasst, die sich in einem Behälter befinden, dessen Form der einer großen Trockenhaube ähnelt und die sich gut den Konturen des Kopfes anpasst. Die von Ihrem Gehirn hervorgerufenen Magnetfelder sind allerdings so klein, dass die magnetischen Änderungen des Erdmagnetfeldes diese Messungen enorm stören können. Aus diesem Grund ist das MEG-System in einer Kammer untergebracht die sie vor elektrischen und magnetischen Störfeldern abschirmt. Die im MEG gemessenen Felder werden anschließend mittels geeigneter Software ausgewertet.

Die Untersuchung erfolgt im Sitzen. Der Kopf befindet sich dabei unter dem eigentlichen Messgerät, die einer Trockenhaube ähnelt. Während der Untersuchung werden Ihnen zwei kurze Videos präsentiert in denen jeweils schnelle Folgen von etwa 600 neutralen und emotionalen Bildern dargeboten werden. Neben neutralen oder gering erregenden Reizen wie Bilder eines Supermarktes (neutral), lachender Kinder (angenehm) oder von Erbrochenem (unangenehm) enthalten sie aber auch stärker erregendes Material. So sind auf der angenehmen Seite beispielsweise Bilder

von Risikosportarten oder Sexszenen zu sehen, auf der aversiven Seite zum Beispiel Kriegsszenen oder Bedrohungsszenen durch Tiere oder Menschen. Die Präsentation jedes dieser Videos wird etwa 6 bis 10 Minuten in Anspruch nehmen.

Auch wenn sich die angesprochenen emotionalen Bilder nicht qualitativ von jenen unterscheiden, welche uns tagtäglich in den Medien präsentiert werden, so möchten wir dennoch sicherstellen, dass die Darbietung dieses Materials keine unverhältnismäßige Belastung für Sie darstellt. Aus diesem Grund möchten wir Sie darauf aufmerksam machen, dass Sie die Messung zu jeder Zeit ohne Angabe von Gründen ohne jegliche Nachteile für Sie unterbrechen können. Natürlich können sie die Messung auch zu jeder Zeit ohne Angabe von Gründen ohne jegliche Nachteile für Sie unterbrechen oder einfach Ihre Augen schließen.

Das MEG-Gerät hält alle für die Sicherheit des Betriebes und insbesondere die Sicherheit von Probanden und Patienten erforderlichen Grenzwerte ein. Es wird in regelmäßigen Abständen gewartet und auf seine Sicherheit geprüft.

Folgende Punkte müssen aber beachtet werden:

Die korrekte Arbeitsweise des Untersuchungsgerätes ist nur in einem Raum gewährleistet, der von allen elektronischen oder magnetischen Störeinflüssen abgeschirmt ist. Folgende Gegenstände stellen wegen ihrer elektronischen oder magnetischen Eigenschaften weitere Störeinflüsse dar:

- Fest im Körper installierte elektronische Hilfsmittel, wie z. B. Herzschrittmacher, Hörgeräte oder Insulinpumpen
- Ferromagnetische Implantate im Körper, z. B. auch Metallsplitter nach Unfällen
- metallische Implantate im Kopfbereich (dies gilt unter Umständen auch bei Zahnersatz)
- Implantierte oder extrakorporale Elektroden oder andere Drähte tragen (Sonden, Stimulatorelektroden, EKG-Elektroden und Anschlusskabel bei Langzeit-EKG, auch nicht angeschlossene Kabel)

Sollten Sie Träger solcher Geräte oder Gegenstände sein, bitten wir Sie, uns vor der Untersuchung darauf aufmerksam zu machen. Diese würden keinerlei Gefahr für Sie darstellen, eine Untersuchung dagegen möglicherweise aber zu sehr stören.

<u>Bitte beachten Sie</u>:
Falls Sie sich entscheiden, an dieser Studie teilzunehmen, bitten wir Sie, uns schriftlich auf diesem Bogen ihr Einverständnis mitzuteilen. Sie können natürlich eine solche Zustimmung jederzeit und ohne Angabe von Gründen wieder rückgängig machen, ohne dass sich Nachteile für Sie ergeben.
Ihre Daten werden auch zum Zweck der Forschung ausgewertet, damit später auch andere Patienten davon profitieren können. Ihr Name und ihre persönlichen Daten unterliegen dabei selbstverständlich der ärztlichen Schweigepflicht.

<u>Wenn Sie noch Fragen haben....</u>
Bitte scheuen Sie sich nicht, weitere Fragen, die Sie möglicherweise im Zusammenhang mit dieser Untersuchung haben, dem Sie untersuchenden Arzt oder anderen Personen unserer Abteilung zu stellen. Sie können sich Ihre Entscheidung gerne in Ruhe überlegen. Sollten Sie zustimmen, bitten wir Sie, die folgende Einverständniserklärung zu unterschreiben.

Einwilligungserklärung

Name der Probandin / des Probanden___________________________

Ich bin über die geplante Untersuchung eingehend und ausreichend unterrichtet worden. Ich konnte Fragen stellen, die Informationen habe ich inhaltlich verstanden. Ich habe alle Fragen des Probandenfragebogens wahrheitsgemäß beantwortet. Ich habe keine weiteren Fragen, fühle mich ausreichend informiert und willige hiermit nach ausreichender Bedenkzeit in die Untersuchung ein. Mir ist bekannt, dass ich meine Einwilligung jederzeit ohne Angaben von Gründen widerrufen kann. Ich weiß, dass die Untersuchung wissenschaftlichen Zwecken dient und die gewonnenen Daten eventuell für wissenschaftliche Veröffentlichungen verwendet werden. Hiermit bin ich einverstanden, wenn dies in einer Form erfolgt, die eine Zuordnung zu meiner Person ausschließt. Auch diese Einwilligung kann ich jederzeit widerrufen.

Münster,____________ ____________________________________
Ort, Datum Unterschrift der Probandin/des Probanden

Münster,____________ _____________________________________
Ort, Datum Unterschrift des Klinikmitarbeiters
